# Self-crafting vegetable snacks: testing the IKEA-effect in children

**DOI:** 10.1108/BFJ-09-2016-0443

**Published:** 2017-06-05

**Authors:** Sanne Raghoebar, Ellen van Kleef, Emely de Vet

**Affiliations:** 1 Strategic Communication Group, Wageningen University and Research Centre, Wageningen, The Netherlands; 2 Marketing and Consumer Behaviour Group, Wageningen University and Research Centre, Wageningen, The Netherlands

**Keywords:** Children, IKEA-effect, Vegetable consumption

## Abstract

**Purpose:**

The purpose of this paper is to test whether the IKEA-effect (Norton *et al.*, 2012) – better liking for self-crafted products than for identical products crafted by others – can be exploited to increase liking and consumption of vegetable snacks in children.

**Design/methodology/approach:**

A between-subjects experiment was conducted at an after school care facility. In total, 86 children aged four to six either crafted a peacock with vegetables or with non-food objects following an example. After the task, children ate snack vegetables ad libitum, and rated their liking for the vegetables and pride in crafting the peacock.

**Findings:**

No significant main effect of the vegetable snack creation on consumption and liking was observed. Also, perceived pride did not mediate the effect of self-crafting vegetable snacks on consumption of and liking for vegetables.

**Research limitations/implications:**

Vegetable consumption did not differ between children who were either simply exposed to vegetable snacks while crafting or those who were crafting the vegetable snacks themselves. The equal consumption might suggest that this is caused by simple exposure, but more research is needed comparing self-crafting and exposure to a condition where there is no initial exposure to vegetables.

**Originality/value:**

Although the IKEA-effect has been demonstrated in adults, this is one of the first studies evaluating the IKEA-effect in children and as a means to increase liking for a generally disliked product in this target group, i.e. vegetables. The IKEA-effect could not be replicated under these more stringent conditions, where the experimental set-up enabled disentangling exposure and crafting effects.

## Introduction

Vegetables are considered as an important part of healthy diets, as evidence suggests that substantial consumption of vegetables can reduce the risk for future chronic non-communicable diseases and obesity (World Health Organization, 2003; Robertson *et al.*, 2004; Leenders *et al.*, 2013; Bazzano *et al.*, 2002). Despite these positive health effects, globally most children do not consume sufficient amounts of vegetables (World Health Organization, 2003); for instance in the Netherlands virtually none of the children aged four to six years meet the recommended daily amount of vegetables (Ocké *et al.*, 2008). Hence, increasing vegetable consumption in children has become an important research topic.

Lately, several studies demonstrated the efficacy of involving children in the process of meal preparation on their subsequent vegetable consumption. A cross-sectional survey among 3,398 Canadian children aged 10-11 indicated that a higher frequency of participating in meal preparation is associated with a higher vegetable preference (Chu *et al.*, 2013). This result is empirically supported by a between-subjects experiment of Van der Horst *et al.* (2014), who showed that involving children aged six to ten in cooking activities significantly increased their subsequent vegetable consumption. Children in the experimental condition prepared a lunch meal with help from their parent, according to a recipe booklet, and children in the control condition were present in the kitchen doing something else while their parent prepared the meal, according to the same recipe booklet. After meal preparation, parent and child consumed standardised plates of their prepared meal together. Results particularly indicated large effects on vegetable consumption, as children in the experimental condition consumed significantly more salad (76.1 per cent) than children in the control condition (Van der Horst *et al.*, 2014).

The positive effect of involvement on vegetable consumption can be explained by a broader trend, that is value creation through co-creation with consumers rather than embedding value creation in the manufacturing process (Vargo and Lusch, 2004; Lusch *et al.*, 2007). For instance, the co-creation of value is pivotal in the concept of the Swedish manufacturer IKEA, in which assembly of the consumer is required for products to arrive (Norton *et al.*, 2012). Same tendencies can be identified in food contexts, as several product successes (e.g. instant cake mixes) may be ascribed to the labour consumers have to put into the preparation of the food (Norton *et al.*, 2012; Dohle *et al.*, 2014).

From this line of reasoning, Norton *et al.* (2012) experimentally demonstrated in adults the existence of a phenomenon which they have labelled as the IKEA-effect: consumers’ increased liking and willingness to pay more for self-crafted products than for identical products made by others. In four experiments they tested the effect with different products that relatively have a more utilitarian nature (i.e. functional, instrumental nature; e.g. IKEA boxes) or a more hedonic nature (i.e. fun, excitement nature; e.g. origami) (Dhar and Wertenbroch, 2000; Norton *et al.*, 2012). Results showed that consumers significantly liked self-crafted products more and were willing to pay more for them. Crafting was done by following a step-by-step instruction sheet, which suggests that the increase in valuation of self-crafted products is not attributable to customisation of the product by the consumer or consumers’ need for uniqueness, as the finalized crafted products were similar. Results also indicated that the effect only emerged when the creation of the self-crafted product was successfully completed, because the effect dissipated when consumers built and then “unbuilt” their creations (Norton *et al.*, 2012). This implies that labour alone is not sufficient for the effect to emerge. However, the specific factors underlying the increase in valuation of self-crafted products are unclear in these experiments. Norton *et al.* (2012) suggested that the drivers underlying the effect vary by the type of product being created. Focussing on the creation of food products, Dohle *et al.* (2014) found similar effects to the IKEA-effect which they have labelled as the “I cooked it myself effect”. In adults, they empirically showed that the mere act of preparing food (high-calorie milkshakes) resulted in a higher liking and consumption amount of the food. Participants in the experimental condition consumed self-prepared milkshakes, prepared according to a given recipe, and participants in the control condition consumed ready-to-drink milkshakes, prepared according to the same recipe. On average, consumption of the self-prepared high-calorie milkshake was 47.9 per cent more than consumption of ready-to-drink milkshakes. However, food preparation in this study could lead to negative health outcomes, as the effect is tested with highly liked calorie-dense food products.

Inspired by the IKEA-effect, the present study aims to evaluate the impact of self-crafting vegetable snacks on vegetable consumption and liking in children aged four to six years, as a novel strategy to promote vegetables in children. The present study replicates and extends previous studies on the IKEA-effect by testing whether the IKEA-effect exists in children and also holds for products that are generally disliked by the target group. Play can be seen as an essential component of the self-crafting task, which is an important tool for children to learn. Through play children engage with their environment from a young age and when they are open to play they get intrinsically motivated, which helps them in acquiring knowledge (Deci, 1975; Burriss and Tsao, 2002; Milteer *et al.*, 2012). Play is essential for a healthy development of young children in terms of cognitive, social, emotional and physical development (Milteer *et al.*, 2012; Burriss and Tsao, 2002). In this study it is expected that playing with vegetables has a positive effect on children’s vegetable consumption. Moreover, in order to isolate the mere effect of self-crafting vegetable snacks on vegetable liking and consumption, the control group will complete an identical crafting task but with non-food items. Further influential aspects are excluded in this study, such as the presence of parents. Furthermore, we aim to explore a potential mediator of self-crafting effects on vegetable liking and consumption, namely, perceptions of pride. In adults it is demonstrated that feelings of pride associated with self-crafted products, in these experiments a LEGO car and IKEA boxes, significantly mediated the effect of creation on valuation. Consumers who crafted products themselves perceived higher feelings of pride and were willing to pay more for their products than consumers who received the same product crafted by others (Mochon *et al.*, 2012). Moreover, people have an innate psychological need to feel competent, which may be fulfilled by the crafting task as children often experience pride after succeeding a new task (Ryan and Deci, 2000; Gagné and Deci, 2005; Tracy and Robins, 2007). For instance, a garden pilot project designed to promote fruit and vegetable consumption in children aged 9-12 showed that the intervention activities generated feelings of pride and ownership, which increased fruit and vegetable consumption and preference in children (Heim *et al.*, 2009). Importantly, children aged four to six years are able to experience feelings of pride and to recognize pride at above-chance levels (Tracy *et al.*, 2005). Hence, it is expected that self-crafted vegetable snacks evoke feelings of pride in children, which subsequently increase their liking and consumption of self-crafted vegetable snacks.

The present research can potentially be used in practice, as the IKEA-effect could easily be implemented in teaching activities or used as a new child-feeding strategy with the goal to enhance vegetable liking and consumption in children aged four to six years. The implementation of the IKEA-effect in practice needs little investments, as only the process of offering vegetables needs to be changed. Furthermore, the present study delivers a scientific contribution to the body of knowledge about the IKEA-effect by investigating the effect in children and with products that are generally disliked by this target group.

## Methods

### Participants and design

In total, 92 children aged four to six years participated in a between-subjects experiment, conducted at six groups of four locations at one after school care facility. Five participants were excluded from the analyses as they did not finish the experiment; for instance parents picked up their children earlier than expected. One participant exceeded the age range and was also excluded from analyses, as the cognitive abilities of children in an older age range are further developed. Data of 86 participants were suitable for analyses (93.5 per cent): 43 participants in the experimental condition and 43 participants in the control condition. The average age was 4.80 years (SD=0.85). Passive informed parental consent was obtained, and parents could object their children to participate in the study. In total two parents did not provide consent for their child to participate in the experiment.

**Figure 1:**
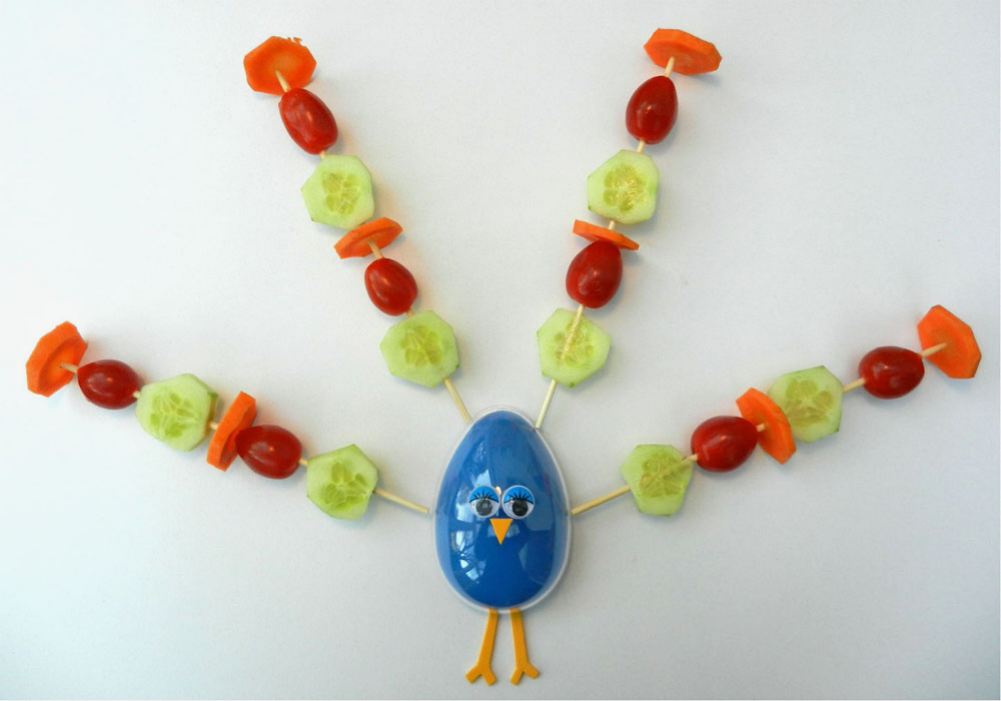
Peacock made of vegetables

**Figure 2:**
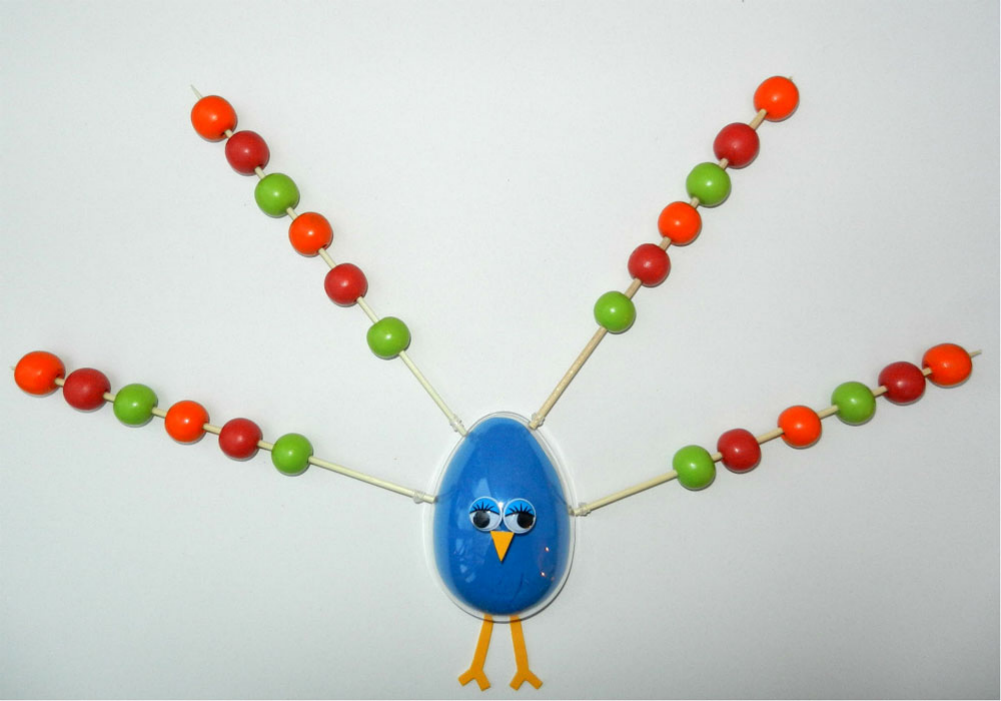
Peacock made of beads

The first 43 participants participated in the experimental condition and the subsequent 43 participants participated in the control condition, in order to prevent contamination between conditions. In both conditions a crafting task was implemented, only crafting material was manipulated. Participants in the experimental condition engaged in a creative task crafting a peacock with snack carrots, tomatoes and cucumbers (see Figure 1), following an example peacock made of vegetables. Participants in the control condition did the same exercise, but with non-food objects (beads) (see Figure 2), following the same example peacock made of vegetables. The crafting task was implemented in both conditions, in order to exclude exposure differences, process enjoyment and customisation as an explanation. All procedures have been approved by the Social Sciences Ethics Committee of Wageningen University and Research.

### Procedure

The tests were completed in small test rooms in the after school care facility between 14.00 p.m. and 18.00 p.m. Two participants at a time completed the study individually in two separate cubicles in the test room, in order to prevent that normative social influences have an impact on the outcome. The tests were conducted verbally since most participants were not competent to read. Before actual testing an additional six participants participated in a pilot study: four participants in the experimental condition and two participants in the control condition. Experimental adjustments were made according to this pilot study. For instance, unipolar five-point scales for measuring perceived effort and perceived pride were changed into unipolar three-point scales, as unipolar five-point scales were too difficult for the participants.

The experiment leaders introduced themselves and the research to both participants and asked for their age and determined their gender. Before the crafting task, participants’ perceived appetite was assessed on a visual bipolar three-point scale. The instructor verbally explained the scale to the participants and tested their understanding of the scale. Subsequently, participants were invited to create a peacock made of snack carrots, tomatoes and cucumbers (experimental condition) or a peacock made of orange, red and green beads corresponding to the colours of the vegetables (control condition), both following an example peacock made of vegetables. This example vegetable peacock was present in both conditions, to rule out the possibility that potential effects on vegetable liking and consumption in the experimental condition were merely due to exposure to vegetables rather than to the crafting task. Further, participants in both conditions received an instruction sheet illustrating the different steps for crafting the peacock with vegetables (experimental condition) or with beads (control condition) and were asked to maintain the specific order and amount of vegetables and beads depicted on the instruction sheets. The instructor verbally explained the different steps to the participants. Each participant had to complete their peacock successfully, therefore no time limit was set. Successful completion meant that each participant had to complete their creation to their satisfaction. After the crafting task, the instructor invited the participants to eat snack vegetables ad libitum from the vegetable peacock crafted by themselves (experimental condition) or crafted by the researchers (control condition). More vegetables were provided when asked, participants were not allowed to take vegetables with them after the experiment. After consumption, participants’ overall liking of the vegetable snack and the individual vegetables were measured. Furthermore, all participants indicated their perceptions of effort and pride in crafting the peacock with vegetables (experimental condition) or beads (control condition). The instructor verbally explained the scales to the participants and tested their understanding of the scale. Finally, the instructor assessed the amount of vegetables consumed.

### Materials

Raw carrots, tomatoes and cucumbers were chosen as food stimuli, as probably most children have previously been offered these vegetables at preschool (Bell and Tepper, 2006). This is important, as children regard unfamiliar vegetables with suspicion, which may negatively affect liking and consumption of vegetables (Wardle and Cooke, 2008). By providing children vegetables they probably already have been exposed to, these familiarity effects were minimized as an explanation. In the control condition beads were used as stimuli in corresponding colours to the vegetables (orange, red and green). In total, 16 vegetable pieces per vegetable sort (experimental condition) or 16 beads per colour were offered in different bowls. Participants needed eight vegetable pieces per vegetable sort or eight beads per colour for crafting the peacock.

### Measurements

#### Consumption amount

Consumption amount was assessed by counting the number of carrot pieces, tomato pieces and cucumber pieces before and after consumption (Wardle *et al.*, 2003). In case a child partially consumed a piece of a vegetable, this was registered.

#### Vegetable liking

Participants’ overall liking of the vegetable snack was measured by asking “do you think the feathers are good or bad?”, rated on a bipolar facial hedonic five-point scale. The scale consisted of five drawings of smileys showing different facial expressions, ranging from 1 (very bad) to 5 (very good). A picture of the vegetable snack was depicted next to the scale, to make sure that children made the connection between the word “feathers” and the actual vegetable snack. Before rating, the instructor verbally explained the scale to the participants. The scale can reliably be used with children in the age range four to six years (Chen *et al.*, 1996; Guinard, 2000).

#### Perceived pride

Participants’ perceptions of pride were measured as a potential explanation for the effect of self-crafting vegetable snacks on liking and consumption. Perceived pride was measured by asking participants “how proud are you of your crafted peacock?”, rated on a unipolar three-point scale. The scale consisted of three pictures of peacocks made of vegetables (experimental condition) or three pictures of peacocks made of beads (control condition). The peacocks on the pictures differed in the number of feathers, ranging from no feathers, to two feathers, to four feathers, designed to, respectively, represent the following responses 0 (not proud at all), 1 (a little proud) and 2 (very proud). Before rating, the instructor verbally explained the scale to the participants. The instructor started with the peacock with no feathers and explained that this peacock is not proud at all as the peacock has no feathers. This was also done for the other two peacock descriptors. The instructor explained that the more feathers depicted on the pictures showed the more feelings of pride.

#### Perceived appetite

Participants’ perceived appetite was assessed by asking “how hungry are you at the moment”, rated on a visual bipolar three-point scale conform and validated by Bennett and Blissett (2014). The scale consisted of three drawings of teddy bears with stomachs showing different degrees of fullness, ranging from 0 (full), to 1 (half full) to 2 (empty). Before rating, the instructor verbally explained the scale to the participants and tested their understanding of the scale by asking the participant to describe their feeling of fullness right after dinner. The instructor showed the participant the “full” teddy bear descriptor and explained that their feeling of fullness would be valued as that teddy bear. This procedure was also used for the participants’ feeling of fullness right before dinner (Bennett and Blissett, 2014). Subsequently, the instructor asked the participant to indicate their feeling of fullness at the moment using the three-point scale.

#### Self-rated effort

Participants’ rating of effort in crafting the peacock was measured by asking participants “how much effort did you put in your crafted peacock?”, rated on a unipolar three-point scale. The scale consisted of three drawings of smileys showing different facial expressions, ranging from 0 (no effort at all), to 1 (a little effort), to 2 (a lot of effort). Before rating, the instructor verbally explained the scale to the participants.

#### Correctness of the crafting task

A dichotomous variable was created (1=peacock crafted with correct amount of materials and in the correct order), which was registered by the instructor.

#### Crafting duration

The instructor registered without knowing of the participants the crafting duration (measured per second).

### Statistical analysis

Data were analyzed using the SPSS statistical software package, version 21.0 (IBM Corp, Armonk, NY, the USA). In all analyses, a significance level of <0.05 was used unless otherwise stated. *p*-values between 0.05 and 0.1 were considered as “marginally statistically significant”. Analysis of variance (ANOVA) and Pearson *χ*^2^ analysis were used to check whether comparability of the conditions was successful in terms of an equal distribution of age, perceived appetite and gender across conditions. Pearson correlation coefficients (denoted by *r*) and Spearman’s correlations (denoted by *r*_*s*_) were performed to assess correlations between the control variables age, perceived appetite and gender, and the dependent variables vegetable liking and consumption. Furthermore, variances across conditions were checked for the variables perceived effort, correctness of the crafting task and crafting duration, using Pearson *χ*^2^ analyses and an ANOVA. ANCOVAs were performed to test whether the dependent variables vegetable liking and consumption were significantly different between the control condition and experimental condition. A Pearson *χ*^2^ analysis was used to determine variance across conditions regarding the variable perceived pride. Perceived pride was further examined using Spearman’s correlations in order to check to what extent the variable predicted consumption and liking in participants in the experimental condition. Finally, a mediation analysis was conducted using Hayes’s PROCESS tool (Hayes, 2013). This tool checked the indirect effect of the intervention on the dependent variables through perceived pride. The significance of the indirect effect was estimated by the Sobel test.

## Results

### Comparability of the conditions

ANOVA and Pearson *χ*^2^ analysis did not show significant differences between the control condition and experimental condition in, respectively, age (*F*(1, 84)=0.40, *p*=0.53) and gender (*X*^2^=0.75, *p*=0.39). In the control condition (41.9 per cent boys) the average age was 4.74 years (SD=0.85, range 4-6) and in the experimental condition (51.2 per cent boys) the average age was 4.86 years (SD=0.86, range 4-6). However, ANOVA did show a significant difference between the control condition and experimental condition in perceived appetite (*F*(1, 84)=7.07, *p*=0.01). Participants who had to create a peacock with beads (*M*=1.33, SD=0.72) had a stronger perceived appetite than participants who had to create a peacock with vegetables (*M*=0.88, SD=0.82). Consequently, perceived appetite was included as a covariate in all analyses.

Spearman’s correlations showed that the control variables gender and perceived appetite did not significantly correlate with any of the dependent variables. However, a modest positive correlation was found between the control variable age and dependent variable vegetable consumption (*r*=0.27, *p*=0.01). Age is predictive for the dependent variable vegetable consumption and is therefore included as a covariate in all analyses.

### Manipulation check

#### Perceived effort

Participants in both conditions reported to put a lot of effort in the task (experimental condition: *M*=1.95, SD=0.21, range 0-2; control condition: *M*=1.91, SD=0.29, range 0-2). Variances across conditions were checked using Pearson *χ*^2^ analysis. The assumptions underlying *χ*^2^ tests were violated, hence the Likelihood Ratio was interpreted instead of the Pearson *χ*^2^. No significant difference was observed between the conditions regarding perceived effort (Likelihood Ratio=0.73, *p*=0.39).

#### Correctness of the crafting task

*χ*^2^ analysis indicated a significant difference between both conditions regarding the variable correctness of the crafting task (Pearson *χ*^2^=5.51, *p*=0.02). Participants who had to craft a peacock with beads showed less difficulty in placing the correct order and amount of beads onto the cocktail sticks (correctness of the task: 81.4 per cent) than participants who had to craft a peacock with vegetables (correctness of the task: 58.1 per cent).

#### Crafting duration

An ANOVA was performed to check whether the completion of the task took a similar amount of time in both conditions. Crafting duration seemed to deviate from a normal distribution, hence data were normalized by taking the natural logarithm of the observations. A significant effect of the manipulation on crafting duration was observed (*F*(1, 84)=12.65, *P*<0.001). Participants who had to craft a peacock with beads (*M*=06:27 minutes, SD=02:35 minutes) crafted a significantly shorter amount of time than participants who had to craft a peacock with vegetables (*M*=09:11 minutes, SD=04:14 minutes).

### Main analyses

#### Consumption amount

On average, participants consumed 17.43 pieces of vegetables (SD=13.08). Almost a fourth of the participants (24.4 per cent) finished their peacock made of 24 vegetables and 17 of these participants consumed additional vegetables (19.8 per cent). The peacock finishers were equally distributed across the control condition and experimental condition (*p*=0.62, Fisher’s Exact Test), just as participants who consumed an additional number of vegetables (*p*=1.00, Fisher’s Exact Test). Only three of these participants finished the entire portion, which consisted of a peacock made of 24 vegetables and 24 additional vegetables. These participants were equally distributed across the control condition and experimental condition (*p*=1.00, Fisher’s Exact Test). In total four participants (4.7 per cent) did not consume any vegetables. These participants were equally distributed across the control condition and experimental condition (*p*=0.62, Fisher’s Exact Test). An ANCOVA was performed to test the effect of self-crafting vegetable snacks on the amount of vegetables consumed, with perceived appetite and age as covariates. No main effect of self-crafting vegetable snacks on vegetable consumption was observed (*F*(1, 82)=1.27, *p*=0.26) (see Table I). Results remained the same when only participants who performed the task correctly (correct order and amount) were included in the analysis (*F*(1, 56)=1.48, *p*=0.23).

#### Liking

On average, participants had an overall liking of vegetables of 4.01 (SD=1.40, range 1-5). An ANCOVA was performed to test self-crafting effects on overall vegetable liking, with perceived appetite and age as covariates. No main effect of self-crafting vegetable snacks on overall vegetable liking was observed (*F*(1, 82)=0.42, *p*=0.52) (see Table I). Results remained the same when only participants who performed the task correctly (correct order and amount) were included in the analysis (*F*(1, 56)=0.32, *p*=0.57).

### Mediator variable

#### Perceived pride

Participants had an average amount of perceived pride of 1.85 (SD=0.47, range 0-2). Variances across conditions were checked using Pearson *χ*^2^ analysis. The assumptions underlying *χ*^2^ tests were violated, hence the Likelihood Ratio was interpreted instead of the Pearson *χ*^2^. No significant difference was observed between the conditions regarding perceived pride (Likelihood Ratio= 0.21, *p*=0.90) (see Table I).

Spearman’s correlations were used to examine to what extent perceived pride predicted consumption and liking in children in the experimental condition. A marginally positive correlation was found between perceived pride and vegetable consumption (*r*_*s*_=0.26, *p*=0.098). No significant correlation was found between perceived pride and vegetable liking (*r*_*s*_=0.24, *p*=0.12).

A mediation analysis was performed using Hayes’s PROCESS tool, in order to test whether perceived pride (denoted by M) mediated the effect of self-crafting vegetable snacks (denoted by X) on vegetable consumption and liking (denoted by Y), with perceived appetite and age as covariates. See Table II for the results. None of the pathways of the mediation model were significant (Sobel test: all *p*’*s*>0.80) and there was no indirect effect as all of the 95 per cent confidence intervals included zero. This means that perceived pride did not significantly mediate the effect of self-crafting vegetable snacks on vegetable consumption and liking.

## Discussion

The main aim of the present study was to evaluate the impact of self-crafting vegetable snacks on vegetable consumption and liking in children aged four to six years, as a novel strategy to promote vegetables in children. The present study replicates and extends previous studies on the IKEA-effect by testing whether the IKEA-effect exists in children and also holds for products that are generally disliked by the target group. Children crafted a peacock with beads or with vegetables and were both exposed to an example peacock made of vegetables. Children who crafted a peacock with vegetables were invited to consume vegetables from their own creation and children who crafted a peacock with beads received a vegetable peacock crafted by others. It was hypothesized that children have an increased consumption of and liking for self-crafted vegetable snacks, rather than vegetable snacks crafted by others. However, results illustrated that self-crafting vegetable snacks did not significantly impact the amount of vegetables consumed and did not significantly increase vegetable liking in children.

These findings are in contrast with other studies that demonstrated that consumers have an increased valuation for self-crafted products compared to identical products crafted by others (Norton *et al.*, 2012; Dohle *et al.*, 2014; Mochon *et al.*, 2012). The main differences between these studies and the current study were that in both conditions we included a fixed crafting task and pre-exposed children to the vegetable snack during this task. With respect to the first point, Norton *et al.* (2012) and Dohle *et al.* (2014) did not include a crafting task of a non-targeted product in the control condition. In these studies significant effects were found of self-crafting products on consumers’ valuation of these products. However, the current study showed that this effect may disappear when also an irrelevant crafting task is included as control. The equally high consumption of and liking for vegetables in both conditions might suggest that this is caused by the experience of performing a crafting task in itself before consumption. Given that the children virtually reported to have put a lot of effort in the task, the consumption and liking in both conditions after performing the task may result from effort justification, licensing the intake of the foods because one “deserves” it (Witt Huberts *et al.*, 2012). With respect to the second point, in the current study children in both conditions were exposed to an example peacock made of vegetables before consumption, while in the experiment of Dohle *et al.* (2014) only participants in the experimental condition were exposed to the food products before consumption. As an alternative explanation, the equally high consumption of and liking for vegetables in both conditions might suggest that this is caused by the mere exposure to the vegetable snack, rather than crafting with vegetables. This explanation is in line with other studies demonstrating that mere exposure to vegetables increased vegetable consumption and liking in children (Hausner *et al.*, 2012; Wardle *et al.*, 2003). However, further research is needed to implement pre-test measures and a third condition that exclude a crafting task and pre-exposure to vegetables in the design of the experiment. In the present study, it was a deliberate choice to exclude pre-test liking measures in the design of the experiment, as children aged four to six years have a short attention span and experience difficulties in task comprehension (Guinard, 2000; Resurreccion, 1998). Likewise, familiarity with the products and food fussiness were not assessed. It would be interesting for future research to test whether the intervention could have different effects for children who differ in their familiarity with the snack vegetables or who differ in food fussiness.

The present study also aimed to explore a potential mediator of self-crafting effects on vegetable liking and consumption, namely, perceptions of pride. In both conditions, almost all children indicated that they were very proud of having crafted the vegetable snack oneself. This seems obvious as in both conditions a crafting task was implemented and children often experience pride after succeeding a new task (Tracy and Robins, 2007). Nevertheless, perceived pride associated with self-crafted vegetable snacks did not significantly mediate the effect of self-crafting vegetable snacks on vegetable consumption and liking in children aged four to six years. This finding is in contrast with previous research that demonstrated that feelings of pride may explain the relationship between creation on valuation (Mochon *et al.*, 2012). However, this study was performed with adults, which could be an explanation for the absence of perceived pride as a mediator in the current study, performed with children aged four to six years. Results may be different for older children, when they move in another developmental stage. This could also explain why the IKEA-effect did not work in children aged four to six years, as comparable studies were performed with older children or adults (Van der Horst *et al.*, 2014; Dohle *et al.*, 2014).

Another explanation of this research could be related to the instruction sheets that were provided to the participants, which is typical for the IKEA-effect. A fixed order and amount of vegetables or beads were outlined on the instruction sheets and children were asked to stick to this fixed order and amount of vegetables or beads in crafting the peacock. Children who had to craft a peacock with beads showed less difficulty in following the instructions and had a shorter crafting duration than children who had to craft a peacock with vegetables. Crafting with vegetables is less common than crafting with beads, which could be an explanation for the significant difference between conditions. Moreover, the instructions could have had a negative influence on children’s feelings of autonomy, as the instructions could have threatened their perceptions of freedom of choice, which subsequently could have affected children’s consumption of and liking for vegetables. Therefore, further research is needed to explore the effect of self-crafting vegetable snacks on consumption and liking by implementing the opportunity for customisation in the experimental design. It is also possible that children who crafted with vegetables already customized the vegetable snack to their preferences, as a counter response to their threatened freedom of choice. A limitation of this research is related to the lack of randomization, as the allocation of groups to the research conditions was not randomised, in order to prevent contamination between conditions. Due to the Dutch after school day care system where group composition is varying on a daily basis, we did not expect that the lack of randomization was too problematic because intraclass correlations between members of the groups of the after school care facility are typically low.

Although the IKEA-effect has been demonstrated repeatedly in adults, this is one of the first studies that tested the IKEA-effect in children and as a means to increase liking for a product that is generally disliked by the target group, i.e. vegetables. The IKEA-effect could not be replicated under these more stringent conditions, where the experimental set-up enabled disentangling exposure and crafting effects.

## Figures and Tables

**Table I:** Differences in consumption amount, liking and perceived pride of participants in the experimental condition and control condition

	Experimental condition		Control condition	
	Mean	SD	Mean	SD
Consumption amount	18.53	14.13	16.33	12.00
Liking (range 1-5)	3.91	1.59	4.12	1.20
Perceived pride (range 0-2)	1.86	0.47	1.84	0.49

**Note:** *n* = 43

**Table II:** Mediation analysis of perceived pride in the effect of the intervention on vegetable consumption and liking

	X→Y (c path)^a^		X→Y (c′ path)		X→M (a path)		M→Y (b path)		Indirect effect (a+b path)		Sobel test	
Outcome variables	*B* (SE)	*p*-value	*B* (SE)	*p*-value	*B* (SE)	*p*-value	*B* (SE)	*p*-value	*B* (SE)	CI 95%	*z*	*p*-value
Total vegetable consumption	3.18 (2.82)	0.26	3.08 (2.83)	0.28	0.04 (0.11)	0.67	2.13 (2.94)	0.47	0.10 (0.44)	(−0.20, 1.23)	0.23	0.81
Overall vegetable liking	−0.21 (0.32)	0.52	−0.22 (0.32)	0.50	0.04 (0.11)	0.67	0.27 (0.33)	0.41	0.01 (0.06)	(−0.05, 0.20)	0.26	0.80

**Note:** ^a^Effect of the intervention without mediator
